# Strategic and flexible LNG production under uncertain future demand and natural gas prices

**DOI:** 10.1016/j.heliyon.2023.e16358

**Published:** 2023-05-18

**Authors:** Noor Yusuf, Rajesh Govindan, Luluwah Al-Fagih, Tareq Al-Ansari

**Affiliations:** a College of Science and Engineering, Hamad Bin Khalifa University, Doha, Qatar; b School of Computer Science and Mathematics, Kingston University London, Kingston upon Thames, UK

**Keywords:** Flexibility, Stochastic modelling, Monte Carlo simulation, Long term contracts, Spot selling

## Abstract

The expectation in global demand for liquified natural gas (LNG) remains bullish in the coming years. Despite the unprecedented impact of the COVID-19 pandemic, and the oil price wars between OPEC and Russia in 2020, causing oversupply and falling prices, the LNG markets continue to demonstrate flexibility and resilience in delivering the needs of different sectors, whilst helping achieve the emissions targets. This is attributed to the high competitiveness amongst LNG producers and suppliers, providing greater confidence for medium-to-long term demand. However, the uncertainties in the current outlook for the return of demand and price growth in the post-COVID period pose difficulty for new liquefaction project investment decisions in the pre-Investment Decision Phase (pre-FID). Accordingly, the consideration of new production and selling strategies is needed in the early design stages of projects to cope with the shift in buyers' sentiments favouring increased reliance on spot and short-term uncontracted volumes, as well as incorporating additional flexibility into long-term contracts. In this study, the economic valuation of the flexible Air Product's AP-X liquefaction technology was investigated considering the modelling of price volatilities, using the mean-reverting jump-diffusion pricing model and Monte Carlo simulation, assuming different demand level scenarios in the high-income Asia Pacific markets based on historical trends. The results clearly demonstrate that embedding flexibility within an LNG production system allows producers and suppliers to diversify selling strategies, and take advantage of the lucrative market conditions when demand and prices increase, and hedge against market risks when demand and prices are low.

## Nomenclature

**Acronyms**
LNG: Liquefied natural gas
FID: Final investment decision
SPAs: Sales and purchase agreements
LTCs: Long term contracts
MTCs: Midterm term contracts
LPG: Liquefied petroleum gases
NGL: Natural gas liquids
TOC: Take or pay contract
NPV: Net present value
ENPV: Expected net present value
APCI: Air Products Chemical Inc.
AP-C3MR: Propane mixed refrigerant process
AHP: Analytic hierarchy process

**Symbols**
CF: Cash flow
t, T: Year, Project life
r: Risk-free interest rate
n, N: Current simulation, total number of simulations
$P_{t}$: Natural gas spot price at time t
DMR: Dual mixed refrigerant process
POCP: ConocoPhillips optimized cascade process
MFC: Mixed fluid cascade process
MCHE: Main cryogenic heat exchanger
MCS: Monte Carlo Simulation
ARIMA: Auto regressive integrated moving average
GBM: Geometric Brownian motion
MCS: Monte Carlo Simulation
HH: Henry Hub pricing system
NBP: New Balance Point pricing system
JKM: Japan Korea Maker pricing system
TTF: Title Transfer Facility pricing system
CAPEX: Capital costs
OPEX: Operating costs
$\theta^{gas},\alpha^{gas}$: Mean-reversion parameters
$\sigma^{gas}$: Volatility of natural gas prices
$\sigma^{jump}$: Standard deviation for jump-diffusion process
$\lambda^{Jump}$: Jump-intensity
$\mu^{Jump}$: Mean for poission process

## 1 Introduction

The importance of natural gas has increased due to more stringent environmental regulations that have been established to reduce the greenhouse gas emissions and limit global warming, following the Paris Agreement in 2015 [1]. Natural gas can be liquefied and economically transported via ships to international markets by cooling it down to −164 °C, reducing its volume to 1/600th of the original volume. This mode of transport has also resulted in improved security in the energy supply, providing the means to overcome political and geopolitical constraints associated with the pipeline supply [2].

On the other hand, the LNG supply chain is complex, involving several parties in production and supply, with varying production capacities and durations. As such, the LNG supply chain consists of four main functions: (a) exploration and production; (b) treatment and liquefaction; (c) shipping transportation; and (d) regasification and distribution to the buyer's local market. Amongst these, the liquefaction process is considered the most capital and energy intensive part of the plant, where up to 40–50% of the capital expenditure of the project is spent [3,4]. In recent years, with the rise in demand for natural gas, several studies have focused their attention on the economic evaluation of the LNG and natural gas supply chains [[5], [6], [7], [8]]. The development of an LNG project is typically divided into two phases: the pre-final investment decision (pre-FID); and the post-final investment decision (post-FID) phases. A project only moves from the pre-FID to the post-FID phase after securing buyers in the global markets through Sales and Purchase Agreements (SPAs) that are convincing for the project investors.

Typically, in long-term supply contracts (LTCs) that could last for up to 20–25 years, a take or pay contract (TOC) is established to obligate the buyers to take pre-determined LNG volumes despite their actual future demand, *i.e.* the volume risks are transferred to the buyer [9]. However, in the last couple of decades, the LNG business has changed notably with the emergence of new projects that have been starting without securing buyers and signing LTCs. According to the International Gas Union (IGU), there was approximately 842.5 MTPA of global liquefaction capacity in the pre-FID phase in 2018 [10]. The IGU further noted concerns about securing long-term markets for the large number of pre-FID projects under the uncertainties of future demand and competition from other projects. Consequently, these challenges significantly influence the decision-making process of LNG projects in the pre-FID phase due to increased economic risks. Thus, there is a need for mechanisms in place to mitigate the impact of such risks by evaluating flexible production and selling strategies based on future scenarios for global demand and LNG price variabilities. The main contribution of this study is a risk-based modelling and assessment methodology, which develops robust production and selling strategies under varying market conditions by embedding flexibility within the early design of LNG production systems. It is envisaged that the proposed methodology could be rapidly applied to essentially evaluate solutions for managing risks in novel scenarios and boost investor confidence during the pre-FID phase.

## 2 Review of flexible systems for LNG production and sale contracts

A large amount of uncertainty is involved in the LNG markets accounting for future market trends is risky as it could impact the success and profitability of investments in LNG production and supply projects. Thus, developing and applying methodologies for quantifying and managing possible risks during a project's life-horizon could mean the differences between its success and failure. The assessment and quantification of both the endogenous uncertainties, arising from technical/project and industry/competitive aspects, and exogenous uncertainties, arising from country/fiscal, market and natural aspects, are essential for the apt response and management of associated risks [11]. This broadly includes the enhancement of flexibility and robustness of system design to shocks.

In real-life projects, investment decisions are usually based on available data and statistical expectations, where demand trends and average prices are considered when designing a system. However, this approach does not necessarily provide optimal results with respect to future uncertainties in prices and demands. This is due to the fact that the value resulting from the joint distribution of uncertainties does not equal the value associated with the average, which can be represented mathematically by Jensen's Law [12]. For example, the net present value (NPV) based on expected demand and prices is generally less than or equal to the expected NPV based on the actual demands and prices. Hence, l evaluation should technically map the entire distribution of uncertainties into the economic performance of a project [12].

### 2.1 Flexibility in industrial systems design

Flexibility in system design can exist either *“on the system”*, where it is associated with flexible managerial decisions [13], or *“in the system”*, where it is associated with the technicality and capacity adaptation within the system [14]. Flexibility in engineering systems has been an emerging area of research in recent years [[15], [16], [17], [18]]. Compared to a standard fixed design, flexibility has been found to improve the net life-cycle performance of an industrial system by 10–30% [18].

Meanwhile, the concepts of financial real options are adopted in the engineering systems [12,19,20], wherein flexibility provides the investor with the ‘right’ but not the obligation to change the system in response to exogenous uncertainties [13]. More specifically, it enables the producer to change the system design parameters efficiently, in terms of cost and time, with the purpose of enhancing its economic and/or environmental performance ( [21]; [19];[22]). Moreover, employing flexibility in the engineering design enhances the overall lifecycle performance, enables efficient resource utilisation during the operational stage, and enables opportunities for continuous sustainable development within the system [19]. The flexibility in engineering design also prevents committing to a specific technology under the uncertainties of its success compared to other competing technologies in the market [23]. Overall, accounting for the flexibility when designing a system minimises the possible undesirable outcomes and financial losses (known as a call option), and maximises the possibility of seizing opportunities and increasing profits (known as a put option) when the market develops.

### 2.2 Liquefaction technologies and their flexibility in LNG production

The liquefaction process is the core of an LNG plant. In the last four decades, licensors developed different technologies based on the mechanical refrigeration process to enhance process efficiency and reduce costs ([24]). The LNG market has been dominated by flexible technologies provided by Air Products Chemical Inc. (APCI). According to the IGU [10], in 2018, 72% of the global LNG capacity was produced in plants adopting APCI technologies, such as propane mixed refrigerant (AP-C3MR) process, AP-X, and AP-C3MR/SplitMR. Additionally, forecasts show that APCI technologies would account for around 68% of the global liquefaction capacity by 2024, followed by ConocoPhillips Optimized Cascade (POCP) process for around 23% of the global liquefaction capacity [10]. According to Al-Mutaz et al. [24], C3MR, AP-X, Dual Mixed Refrigerant (DMR), Mixed Fluid Cascade (MFC), and ConocoPhillips Optimized Cascade (POCP) processes are the most common technologies in the LNG market. Each of these processes has multiple options to influence their production capacities. On the other hand, the selection of a baseline natural gas liquefaction technology is a decision-making problem. Different models and tools were reported in the literature for assessing different LNG regasification technologies at the receiving terminals wherein similar models could be used for selecting a liquefaction technology [25,26].

Some studies in the literature further reported the relationship between the flexibility of LNG production systems and uncertainties in the buyers’ markets[[6], [20], [27], [28]]. Hu and Cardin [19] developed a framework for generating flexibility in the design of an engineering system to improve lifecycle performance. The framework consists of four-main steps: baseline performance model; dependency and uncertainty analysis; identifying opportunities to embed flexibility; and validating the methodology by comparing the flexible design with the benchmark design.

Cardin et al. [27] presented an innovative approach for embedding flexibility in engineering systems to improve the expected value of large-scale capital-intensive projects. The authors studied the drivers in the value of flexibility and considered different combined effects of uncertainties, economies of scale, learning, and geographic distribution on the project, in addition to the benefits of adopting and considering flexibility in the early design stages (pre-FID) of the project. They further applied the approach to design an LNG production system for a specific market.

Furthermore, Xia et al. [20] reported a four-step framework as a decision-making tool to design a flexible LNG system in Singapore considering the local market uncertainties. The main objective of the framework is to evaluate the optimal flexible design configuration based on uncertainty, stochastic, and sensitivity analyses. The authors also studied different centralised and decentralised designs. In addition, uncertainties in annual volatility, variations in annual demands, and variations in other input parameters were considered in their study.

Previous studies considered the overall design configuration of an LNG plant rather than studying a particular LNG technology when designing a flexible production system. A study by Hönig et al. [6] analysed the technological and economic characteristics of an LNG production system in Europe for the utilisation of LNG as a fuel for vehicles within the European markets. The study particularly focused on the globally common C3MR technology for LNG production and the decentralisation of LNG production systems around Europe. An engineering model was also implemented in Aspen HYSYS software where the results were used to calculate the NPV of the project by considering forecasted LNG demand, LNG prices, electricity prices, feed gas prices, and CO_2_ emission allowance prices using a time-series forecasting model called Auto Regressive Integrated Moving Average (ARIMA).

### 2.3 LNG pricing and contractual structures

Natural gas and LNG markets are regionally segmented, where different pricing systems are used in different regions [[29], [30], [31]]. The LNG business started typically with long-term contracts (LTCs) with strict clauses to link producers and buyers to minimising investment risks. However, this approach has been changing with the emergence of portfolio players and the liberalisation of pricing systems in the US (Henry Hub) and European markets (Title Transfer Facility and National Balancing Point), shifting the markets away from classical oil-indexed LTCs towards shorter-term contracts and spot selling. In 2018, the global non-long-term LNG trade (i.e. volumes traded on spot or under contracts with less than two years of duration) increased by 2% from 2017, where the total share has represented 31% of the global LNG trade [10]. Consequently, exporters can only guarantee a sustained dominant position in the future by coping with the market changes and buyers' preferences in major importing markets, such as the Asia Pacific, by diversifying the selling strategies; using pricing systems to hedge against market fluctuations; and looking for favourable market conditions.

For investment decision-making under uncertainty, pricing models such as Geometric Brownian Motion (GBM) and mean-reversion models are widely used, particularly to represent natural gas spot prices, oil prices, and ethanol prices [[32], [33], [34]]. Ranjbari et al. [33] reported that natural gas spot prices had been considered mean reverting, in which the prices frequently move up and down around a long-term equilibrium mean natural gas price. Consequently, natural gas spot prices can be modelled using GBM with a mean-reversion factor. In addition, accounting for the jumps in prices resulting from unexpected events or shocks when modelling natural gas spot prices is essential for better prices representation, where a jump-diffusion model is then added to the mean-reverting GBM model [32,35].

Additionally, Monte Carlo simulation (MCS) is used for simulating the scenarios of a system by considering the joint properties of probabilistic distributions of uncertain variables [12]. Real-world complex systems with potentially large investments can be modelled using MCS on the basis of these distributions, continuous or discrete, to quantify the uncertainties, and associated risks, impacting the NPV estimations [36]. Moreover, MCS has the added advantage of simplifying the complexity and mathematical limitations of closed-form analytical solutions [36].

### 2.4 Brief technical analysis of the LNG price fluctuations caused by the ongoing COVID-19 pandemic

The global LNG prices have decreased since the start of the pandemic due to the reduction in demand, mainly from industries and households, leading to oversupply in the market. In 2020, the Henry Hub (HH) natural gas average price decreased by around 21% relative to 2019, reaching $2.03/MMBTU. Prior to the pandemic, the prices had declined due to a mild winter in 2019 that naturally led to lower natural gas demand. During the pandemic in June 2020, the HH natural gas spot prices dropped to $1.63/MMBTU, the lowest monthly average in decades. The prices, however, started to recover in the second half of the year after decreasing natural gas production. The U.S Energy Information Administration (EIA) projected an increase in the average HH natural gas spot price to $2.89/MMBTU in 2021 after further recovery of markets and business activities [37]. In fact, the pandemic is not the first shock which impacted the natural gas market. It had previously experienced a similar behavior in prices in 2009, following the 2008 global recession where behaviour the HH prices dropped by around 56%. Additionally, in 2012, the HH prices fell by 31%, compared to 2011, due to oversupply and a mild 2011 winter, where different projects postponed their decisions. The markets recovered in 2014 and 2015, where the HH prices averaged $3.73/MMBTU and $4.37/MMBTU, respectively. Based on these facts, the level of impact of the pandemic on energy prices was indeed relatively lower when compared to the other major shocks in the past. However, one obstacle in the pandemic is owing to the challenges posed by working at high operational costs and low revenues, not covering production costs in some of the projects. As a result, the low price risk attributed to the delayed start of new projects or expansions, until the markets recover from the shock, especially for countries with high operational costs, such as Australia and the U.S.

### 2.5 Limitations and objectives

The limitations identified in previous studies are mainly attributed to the lack of a link between the flexibility in certain production systems with uncertainties in buyers’ international markets. Moreover, previous studies did not account for the technical and technological aspects of flexibility within LNG production systems. Thus, the work presented herein is novel in that it integrates the technical aspects of flexibility using process system engineering principles together with the concepts and tools from financial engineering to enable the producer to profitably vary productions based on volatile market conditions. Towards this end, the specific objectives of the proposed methodological framework include:

(i) assess historical demand and prices data subjected to systemic shocks, including the impact of major global events, such as Hurricane Katrina and Rita in 2005; the financial crisis in 2008, and the COVID-19 pandemic in 2020-21;
(ii) develop flexible modes of planning and response by producers and suppliers working with market uncertainties from the early stages of project design, including evaluating the value of a flexible production train designs;
(iii) devise and assess selling strategies and customer portfolios, with particular focus on the Asian markets, using forecasting models for future HH natural gas prices.

The framework aims to assess the net ‘project value’ associated with the flexibility of the chosen technology by considering both the technical and economic aspects with respect to the performance in final customer markets. In view of this, the framework consists of five main steps: selection and economic evaluation of a baseline LNG production technology deterministically; economic evaluation of the technology under uncertain LNG selling price; economic evaluation of the technology under demand scenarios and uncertain prices; proposing a hedging strategy through diversifying the selling strategy; and finally evaluating the robustness of the proposed selling strategy under different input parameters.

Furthermore, considering the state of Qatar as a case study, despite the low upstream costs that supported the local LNG industry, the emergence and expansion of new projects worldwide have been impacting Qatar. This has primarily steered the current tendencies of buyers in committing against long-term contracts, which in turn raised financial risks associated with the investments for new LNG projects in Qatar. Consequently, using the methodology developed in this study, the profitability of an LNG production system in Qatar producing LNG to high-income Asia Pacific markets under demand and price uncertainties was also investigated.

## 3 Available data and assumptions

In the integrated decision methodology proposed in this study, only the strategic scenario-based planning of an LNG project was assessed considering the flexibility of production to cope with uncertain demand and prices. As such, detailed tactical planning associated with seasonal demand and inventory management was not considered in this investigation. A case study analysis was performed for a hypothetical project involving a single-train brownfield LNG production plant operating in Qatar with an expected lifetime of 25 years. The project is dedicated to using varied selling strategies in different markets, with a special focus on high-income Asia Pacific markets, including China, Japan, Korea, and low-income markets, such as Pakistan and India. In addition, the correlation between the demand for other energy resources and demand of natural gas in target markets was not investigated.

The following data and assumptions were primarily utilised from the literature for the purpose of demonstration of the methodology:

• Steuer [38] reported that the capital costs for previous brownfield LNG production plants in Qatar ranged between $660-$760/tpa. Hence, a mid-value of $710/tpa was considered. Furthermore, the anticipated gas supply costs and liquefaction costs for the year 2025 are $2.0/MMBTU and $1.69/MMBTU, respectively [38];
• the risk-free interest rate was considered to be 10% [39];
• a project's lifetime of 25 years, including 5 years of construction and 20 years of production;
• the capex distribution throughout the construction period was considered as 6% in the pre-FID phase (year 0); 5% in year 1; 29% in year 2; 33% in year 3; 25% in year 4; and 3% in year 5 [3].
• scenario-based approach representing the LNG demand uncertainty in high income Asia-Pacific markets was assumed to be at three levels: 7.8 MTPA as the high demand case; 6 MTPA as the median demand case; and 5 MTPA as the low demand case. The project's profitability was additionally assessed through selling the remaining capacity to the low-income markets.

Despite the impact of COVID-19 outbreak on the global LNG trade in 2020, LNG demand in China and India recovered relatively faster owing to the resumption of their industrial activities, corresponding to an increase in imports by 11% [40,41]. In the short term, however, it is expected that the global markets would need anywhere in the range of one to four years to return to the pre-COVID-19 levels [42]. As such, the forecasts show that the impact of COVID-19 pandemic could show its effects on the global LNG trade until 2025 when the liquefaction capacity would outpace the growth in the LNG demand [41]. However, the LNG demand in emerging Asian markets is expected to further increase at a faster pace, especially following the announcement of targets for decarbonising the Chinese sectors to become carbon neutral by 2060. Consequently, the Asian markets are the most resilient to global changes in terms of LNG trades. On the other hand, selling in the Asian markets would also create competition between different LNG producers. Hence, a scenario-based approach that considers uncertain future demand levels was followed to assess the profitability of selling LNG in the Asian markets under price uncertainties, specifically impacted by the contrasting effects of customers’ demand levels and the competitive contractual structures offered by the producers.

The contracted selling price to high-income Asia Pacific markets under LTCs was assumed at $8.76/MMBTU (for 2025), as estimated by Steuer [38]. Whilst the HH natural gas spot price was used to calculate sales of LNG in the spot market, with an initial forecasted price of $3.06/MMBTU for 2025 [38]. Moreover, this was the price at which it was assumed that the LNG could be potentially sold to the low-income markets. Incidentally, this price was found to be even lower than the reported historical spot prices for selling in the low-income markets, thus assumed to be best suited as the worst possible scenario of the spot price level in terms of the profitability of the project.

**Fig. 1:**
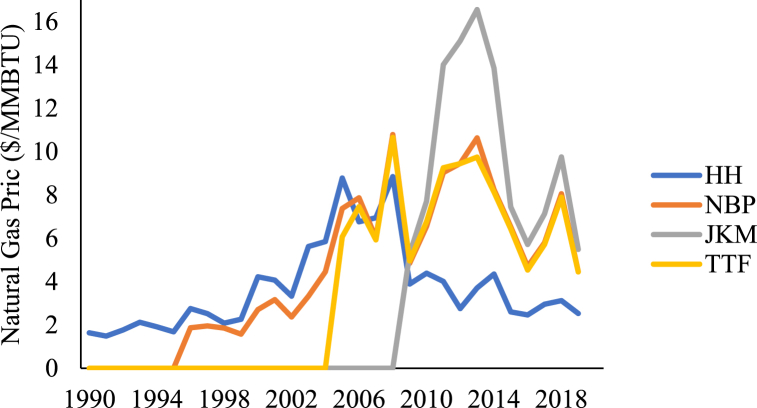
Natural gas prices from 1990 to 2019.

Meanwhile, it is also important to note that there are different regional natural gas pricing hubs for LNG imports and/or exports. The US Henry Hub (HH), UK New Balance Point (NBP), and European Title Transfer Facility (TTF) pricing systems are considered the most liquid and oldest established hubs compared to Japan Korea Maker (JKM) pricing system. Fig. 1 shows the historical data available for the HH, NBP, TTF, and JKM pricing systems from 1990 until 2019 [43]. Only the HH prices are available from 1990, whereas the NBP was established in 1996 and TTF in the early 2000s. Consequently, this study considers the HH natural gas spot prices due to its relative maturity and favorability in the global markets. Lucheroni & Mari [35] had previously derived representative price modelling parameters, including mean reversion, volatility, and Poisson jump statistics, from the HH pricing system dataset for the period of 1990–2013. These values were specifically adopted for the stochastic price simulations in this study to estimate the net present value (NPV) distributions under the different demand scenarios assumed for the Qatar-based LNG project.

## 4 Methodology

**Fig. 2:**
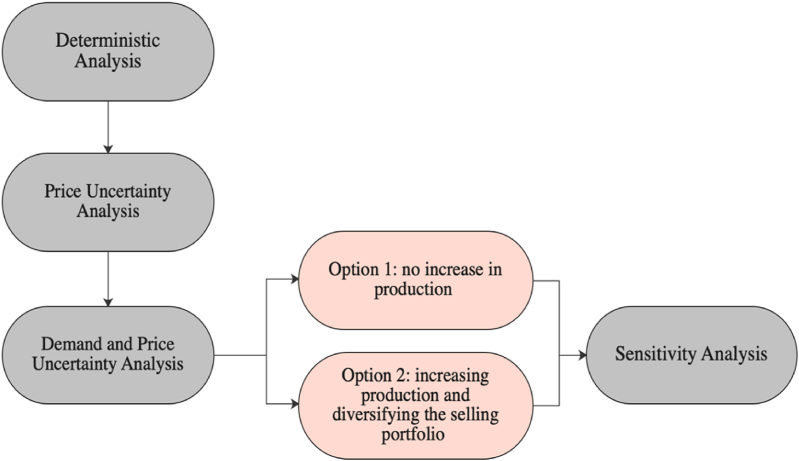
Methodological framework for assessing the flexibility of an LNG production system.

A four-step methodology was developed to assess the value of flexibility in an LNG production technology for enhancing its responsiveness to the demand and price changes in the high-income Asia Pacific markets by considering the stochastic analysis of simulated spot prices. It further analyses the impacts of uncertainties of the HH natural gas spot prices on the profitability of the project. The methodology consists of: (1) selection and economic evaluation of a baseline LNG production technology under deterministic prices and demand; (2) stochastic economic evaluation of the technology under the HH natural gas prices and fixed demand; (3) stochastic economic evaluation of the production system under different demand scenarios subject to real-options strategy based on two decisions of expanding the production in option 1 and not expanding the production in option 2; (4) evaluation of robustness of the decision taken in option 2 under different input parameters. Fig. 2 illustrates the methodological framework for the LNG production system case study in Qatar dedicated for the high-income Asia Pacific region as a primary market.

### 4.1 Deterministic selection and economic evaluation of a baseline production technology

In the first step, the most feasible technology was selected based on specified criteria and evaluated deterministically under the average LNG demands and prices. Additionally, any uncertainty associated with the final market was not considered.

#### 4.1.1 Selection of a baseline technology

LNG process technology selection is a critical step that is considered in the pre-FID phase of the project. In the selection criteria, different aspects were considered to account for the feasibility of the process to meet the objective of embedding production flexibility within the system. In this analysis, the most industrially matured LNG technologies were considered, namely: (a) C3MR by APCI; (b) DMR by shell; (c) AP-X by APCI; and (d) MFC by Linde. Despite the maturity of the POCP technology, it was excluded here since natural gas liquids (NGLs) and liquefied petroleum gases (LPGs) are removed within the liquefaction process. Whilst in this analysis, LPG and NGL were assumed to be removed before the production system.

Using Microsoft Excel, the Analytic Hierarchy Process (AHP) was specifically utilised to select the baseline liquefaction technology based on the identified criteria. In fact, all alternatives are efficient and capital intensive. Hence, the selection of a liquefaction technology wasbased on criteria that primarily consider the flexibility of the technology in terms of operation and variable production capacity; the ease of operation; the economies of scale; and the reliability of the process and its equipment [24]. A two-level decision hierarchy was developed upon identifying the criteria and process alternatives. First, pairwise comparisons were considered for numerically prioritizing the importance of each criterion concerning another on a scale of 0–4. While in the second step, each process alternative was numerically evaluated based on its performance with respect to the criterion. Finally, the calculated weights' accuracy was assessed using the random consistency index [44]. AHP does not necessitate any authentic information sets. Hence decision-making can be subjective and influenced by the judgment of the decision-maker.

#### 4.1.2 Deterministic economic evaluation of the selected baseline design

The main drivers for the economic performance of an industrial project are demand, costs, interest rate, and selling prices of products. Uncertainties in any of these variables would influence the project's profitability. In this step, only the deterministic (average) values were assumed for assessing the economic performance of the baseline design. The standard indicator for the evaluation of economic performance is the overall profitability of a project throughout its lifetime, called the net present value (NPV), represented by Equation (1):

(1) $$NPV=Initial\;investment+\sum_{t=1}^{T}\frac{CF_{t}}{{\left(1+r\right)}^{t}}$$

where the initial investment is the Capex throughout the installation and construction period of the project. CF_t_, the cash flow, is the amount of cash that comes into and out of the project at time t multiplied by the discount factor to account for time value of money, while *r* is the risk-free interest rate. The following assumptions were considered for calculating the NPV:

• The main market for selling LNG is the high-income Asia Pacific market (for long-term contracts, or short-term contracts and/or spot sales indexed to the HH natural gas spot prices)
• all input parameters were assumed to be deterministic and fixed at the average values
• all production and selling prices were indexed to the year 2025, which is assumed to mark the start of production
• all capital and operational costs were indexed to the year 2019, which is assumed to be the pre-FID phase of the project
• only LNG revenues were considered, NGL/LPG revenues were assumed to offset costs in the upstream part of the project. Moreover, shipping costs/revenues were not considered.
• LNG indexed to the HH natural gas spot prices is sold based on Equation (2) [45]:

(2) $$LNG\;price\;\left(\frac{\$}{MMBTU}\right)=1.15HH+liquefaction\;fees$$

### 4.2 Economic evaluation under fixed production and uncertain prices

Several market attributes influence the stochastic behavior of natural gas spot prices such as demand fluctuations, competitiveness with other energy resources, and oil prices. In order to model these uncertainties, the natural gas spot pricing models were investigated to perform stochastic simulation, and the subsequent determination of the corresponding NPV distribution. The dynamic pricing model used in this study is called the mean-reverting Geometric Brownian Motion (GBM) with jumps (the jump stochastic process which in turn follows a Poisson distribution), given by:

(3) $$dlog\left(P_{t}\right)={(\theta }^{gas}-\alpha^{gas}\;\log\left(P_{t}\right))dt+\sigma^{gas}dW+JdN$$

where, P_t_ is the Henry Hub natural gas spot price at time t; $\theta^{gas}$ and $\alpha^{gas}$ are the mean-reversion parameters; $\sigma^{gas}$ is the volatility of Henry Hub natural gas prices; W is the standard Brownian motion (also known as a Wiener Process), with zero mean and a variance of t; and N is the jump-diffusion process (Poisson process), with a jump amplitude of J and intensity $\lambda^{Jump}$. Poisson Process is a discrete distribution with a mean of zero and a standard deviation $\sigma^{Jump}$. Moreover, N and W are considered independent processes. The model parameters were obtained from literature based on data from 1990 to 2013, as indicated in Table 1 [35].

**Table 1:** Geometric Brownian Motion and jump diffusion process model parameters (Lucheroni and Mari, 2018).

Parameter	Notation	Value
Mean-Reversion parameters	$\theta^{gas}$	0.0432
	$\alpha^{gas}$	0.0292
Jumps intensity	$\lambda^{Jump}$	0.2542
Standard deviation for Poisson process	$\sigma^{jump}$	0.1258
Volatility of gas prices	$\sigma^{gas}$	0.0737
Mean for Poisson process	$\mu^{Jump}$	0.0100

The mean-reverting GBM describes the normal fluctuations in natural gas prices only, whilst the jump diffusion model describes the abnormal fluctuations due to shocks caused by different factors such as shocks in oil prices, sudden increase in demand, seasonality, disruptions in production, or global pandemics, such as the COVID-19. The model parameter values were considered to be efficient in terms of capturing these shocks since within the analysed period (1990–2013), the HH natural gas prices were subject to historical shocks that caused the prices to decline, such as the abnormal events that resulted in price spikes owing to the lack of supplies to keep pace with demand in 2001–2002; hurricanes Katrina and Rita in 2005; and, most notably, the global recession in 2008–2009 that led prices to slump.

The stochastic model represented in Equation (3) was implemented using a Monte Carlo Simulation (MCS) to model the possible future price path expectations for the HH natural gas spot prices [46]. MCS is mainly used to approximate the probability distribution of all possible random variables to analyse the uncertainties of a certain scenario or a system. In this analysis, Python programming language in Visual Studio Code enabled by Anaconda software was used to create a large sample size of 10,000 price paths using Monte Carlo Simulation (MCS). The simulated LNG prices were simultaneously used to calculate 10,000 NPV estimates, using Equation (1), at fixed capital and operational costs, risk-free interest rate, and project lifetime, thereby obtaining a histogram to approximate the NPV probability distribution. The expected net present value (ENPV) was subsequently calculated using Equation (4):

(4) $$ENPV=\frac{1}{N}\sum_{n=1}^{N}NPV_{n}$$

where the ENPV is the sum of NPVs (from n = 1 to 10,000) divided by the total number of simulated NPVs given by MCS. The main objective is to compare the stochastic NPV case with the deterministic case using the ENPV measure in order to assess the expected project's profitability under the HH natural gas prices' uncertainties. In addition, the profitability of the project when selling at a fixed LTC price throughout the project's lifetime was also compared with that of selling on the spot market, which is linked to the HH natural gas spot prices to investigate the influence of stochastic prices on the project's profitability.

### 4.3 Economic evaluation under multi-level demand scenarios and uncertain prices

This step assessed the value of flexibility of the selected LNG production technology using two analysis options, considering multi-demand level scenarios and price uncertainties. Throughout the project's lifetime, the decision-maker has the option to increase the production capacity based on market conditions. Hence, this analysis is essential to investigate the capabilities of a flexible production technology in capturing favourable market conditions by diversifying the selling strategy and minimising risks when the demand or spot prices are low. Moreover, flexibility in production is an approach to convince decision-makers to rely less on the classical oil-indexed LTCs and to design a flexible selling strategy. Initially, the technical aspects and limitations of changing the production level in the selected technology were investigated. Then, the profitability of the project was assessed under the stochastic HH natural gas prices, considering different demand scenarios and selling strategies (options).

**Fig. 3:**
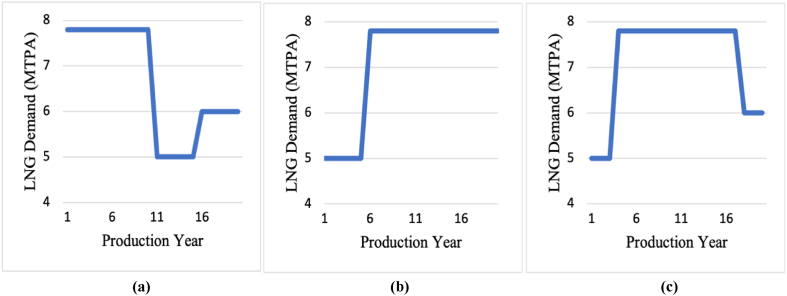
Demand Scenarios in high-income Asia Pacific markets: (a) Scenario 1; (b) Scenario 2; and (c) Scenario 3.

In this analysis, 65% of the production capacity (5 MTPA) was assumed to be contracted to high-income Asia Pacific markets for 20 years, whilst the remaining 2.8 MTPA were assumed to be sold on the spot markets, or short/mid-term contracts when the demand increases either in Asia Pacific or the low-income markets. The project was assumed to utilise the AP-X technology to capture any high demand opportunities in the early stages of production, in which it could be expanded to operate at full capacity, if there is a meaningful increase in demand with high profitability. Alternatively, if there is insufficient increase in the demand, or if the spot prices are low, the investor has the right not to expand the capacity. Three demand scenarios, illustrated in Fig. 3(a)-(c), were studied considering different production options and selling mechanisms such as LTCs, short/mid-term contracts, and spot selling, where each selling mechanism has its own pricing system. The demand levels represented in these scenarios are for the primary Asia Pacific market, the largest LNG market with the greatest premium.

In the first 10 years of production in scenario 1, as illustrated in Fig. 3(a), the demand in the Asia Pacific market is high due to a major shift to cleaner fuels, which can be utilised through increasing the production to the maximum in order to meet the demand. In the following 5 years, the demand could then drop due to competitiveness in the market to increase slightly again by 1 MTPA in the last 5 years of production due to industrial needs. In the first 5 years of production in scenario 2, as illustrated in Fig. 3(b), the market is competitive due to the high added LNG volumes from competitive suppliers. Hence, only the 5 MTPA of contracted LNG will be produced. The project can be expanded to the full capacity starting from year 6 till the end of the project when the market develops. Whilst in scenario 3, as illustrated in Fig. 3(c), the market is competitive in the first 3 years of production to increase starting from year 4 to year 17, with the decline of LNG volumes from competitive projects. Hence, the project can be expanded to sell uncontracted LNG under the HH natural gas spot prices. In the last 3 years of production, the demand is anticipated to decrease to 6 MTPA due to shifting to alternative fuels or renewables in high-income Asia Pacific markets. As such, the reason behind demand uncertainties could be due to seasonality, competitiveness with other energy resources, competitiveness with LNG volumes coming from other exporters, or recession at the importing countries impacting their tendency to buy LNG.

On the other hand, if the increase in demand in the primary market is less than 2.8 MTPA, i.e. 6 MTPA only, the decision makers hold the option to (a) produce only contracted capacity and to not cover the increase in demand in Asia Pacific markets; or (b) to operate at full capacity and cover the increase in demand in Asia Pacific markets by selling LNG based on HH prices and sell the remaining production capacity to low-income markets at lower assumed fixed prices. The economic performance of both options was analysed in this step. The assumptions used for developing the scenarios and selling strategies are summarised below:

• the assumed anticipated demand levels are 5 MTPA, 6 MTPA, and 7.8 MTPA;
• long-term contracted LNG volumes were assumed to be sold at a fixed price of $8.76/MMBTU throughout the project's lifetime, while LNG to be sold to low-income markets at a fixed price of $3.00/MMBTU;
• the price of LNG of MTCs and spot sales were assumed to be indexed to HH spot price;
• revenues from selling LNG were estimated deterministically and stochastically by using the mean reverting GBM jump diffusion model and MCS;
• if there is an intermediate level of demand between 5 MTPA and 7.8 MTPA, considering technical limitations, LNG production is produced at the full capacity of 7.8 MTPA, wherein the excess demand was assumed to be sold to low-income markets, such as India and Pakistan.

### 4.4 Evaluating the robustness of the diversified selling strategy under varied input parameters

In the final step of the methodology, the sensitivity of economic performance in option ‘b’ (with full production capacity expansion) was assessed under different variabilities of input parameters, such as high capex, low capex, an interest rate of 15%, and varied HH initial price. The subsequent impact on the ENPV and NPV probability distribution for each sensitivity assessment case was subsequently analysed. Incidentally, a more stable ENPV under changeable input parameters indicates greater robustness of the scenario to any variabilities.

## 5 Results and discussion

Employing flexibility within LNG production systems is an active mode of response to manage exogenous uncertainties in the final market. The methodology considers a certain production technology to embed production flexibility and assess the value of flexibility of a diversified selling strategy. The results of each step are presented and discussed in the following sub-sections.

### 5.1 Step 1: deterministic analysis of AP-X technology

**Fig. 4:**
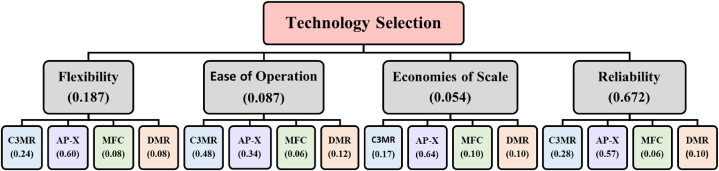
AHP results for selecting a baseline technology.

Initially, the baseline design was selected from amongst the most industrially matured LNG production technologies in the market. Using the AHP technique, the AP-X technology scored the highest aggregate of all criteria that were considered, as illustrated in Fig. 4, with a total score of 0.558. The AP-X technology, licensed by APCI, is an extension of the C3MR process and consists of three cooling cycles: the pre-cooling cycle using propane as a refrigerant; the liquefaction and sub-cooling at the Main Cryogenic Heat Exchanger (MCHE) using mixed refrigerants; and the final sub-cooling in the nitrogen expander [47]. The AP-X technology was specifically designed to increase the production capacity of a C3MR process from 5 MTPA to 7.8 MTPA by adding a single efficient nitrogen expander and adjusting the flowrate of the mixed refrigerant in the second cooling cycle [48].

The economic performance of the AP-X technology was evaluated deterministically by assuming a dedicated full capacity to high-income Asia Pacific markets throughout the 20 years of production based on the two assumed selling mechanisms (LTCs and spot selling) under fixed pricing model parameters. When selling the full capacity based on LTCs throughout the project lifetime at a fixed price of $8.76/MMBTU, the deterministic NPV is estimated as $5.82 billion. This value is profitable and theoretically justifies the reason behind using LTCs for securing sales to hedge against potential market risks. However, in real life scenarios, LNG prices are re-evaluated and adjusted every 5 years of the lifetime of the contract, based on the market's performance. Moreover, considering the changes in the contractual structures in today's market, it is almost impossible to guarantee contracting the full production capacity of a mega project. Hence, the economic performance of the technology was also evaluated deterministically by assuming selling the full capacity in the spot market at an average HH natural gas spot price of $3.06/MMBTU. The estimated NPV in this case is $-1.31 billion, indicating a loss. The latter approach was also tested stochastically by considering the uncertainties in the HH natural gas spot prices throughout the project lifetime in the second step.

### 5.2 Step 2: expected NPV under stochastic Henry Hub prices and fixed production

The deterministic NPV does not capture any demand and HH natural gas prices uncertainties. Therefore, in this step, stochastic simulation modelling for the HH natural gas spot pieces was implemented to estimate the ENPV, considering a constant production level. Fig. 6 illustrates the NPV distribution for a selling strategy for 7.8 MTPA of LNG produced by an AP-X technology under the stochastic HH natural gas spot prices simulated 10,000 times using MCS with the mean-reverting GBM jump-diffusion model. The simulation resulted in an ENPV of $-3.58 billion, which is a loss and indicates higher unexcepted risks involved in the project when dedicating the full capacity to be sold on spot. It further indicates that the HH natural gas spot prices, with greater probability, tend to fall below the initial forecasted price $3.06/MMBTU, through the project's lifetime. Hence, on average, dedicating the full capacity for spot selling is not economically viable to cover the production and liquefaction costs and provide the desired return on investment. On the other hand, Cardin et al. [27], reported the same approach for an LNG production facility under uncertain demand, wherein the calculated ENPV was less than the deterministic NPV. This implies the importance of considering the whole distribution of uncertainties to value the project properly.

**Fig. 5:**
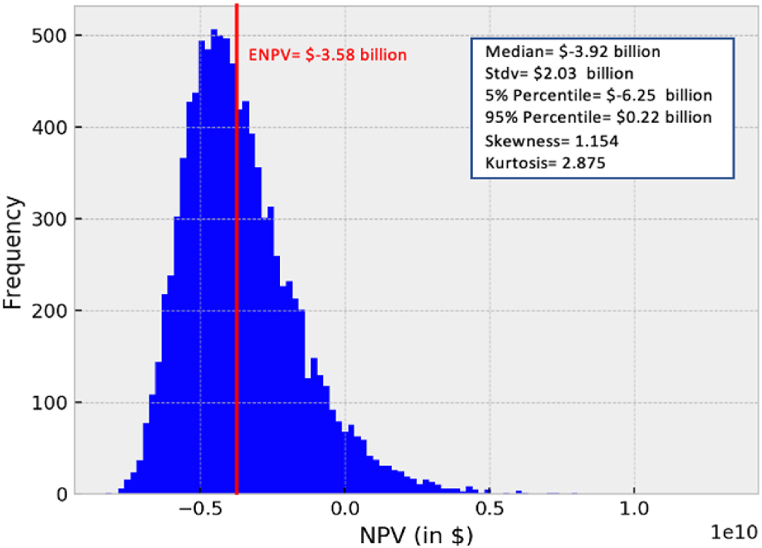
NPV distribution of an AP-X technology with initial forecasted Henry Hub natural gas spot price of $3.06/MMBTU.

Although the HH natural gas spot pricing system is the most mature amongst the different spot pricing systems, it is still highly volatile, as far as the long-term economic feasibility of a project design is concerned, as indicated in Fig. 5. This indicates the need for either minimising the operational costs of the project or to employ a diversified selling and flexible production strategy to hedge against market fluctuations and cope with the transformations in the contractual structures. The latter option is considered in the third step since the production and liquefaction costs of an LNG project in Qatar are the lowest amongst the worldwide projects [38,45]. Hence, multi-demand scenarios and uncertain prices analysis aim to assess the project's economic performance under different conditions by linking the flexible production approach with a diversified strategy.

**Fig. 6:**
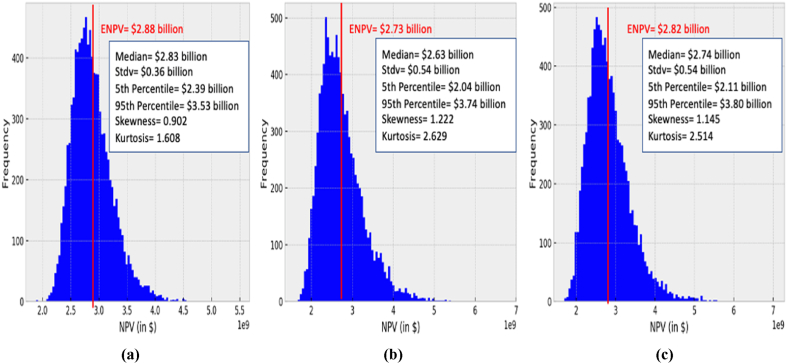
NPV distribution of an AP-X technology with initial forecasted Henry Hub natural gas spot price of $3.06/MMBTU under different demand scenarios for selling in high income Asia-Pacific markets only (Option 1: deciding to not increase the capacity): (a) scenario 1, (b) scenario 2, and (c) scenario 3.

### 5.3 Step 3: the influence of the stochastic behaviour of HH prices on ENPV of scenarios

The selected AP-X technology produces 7.8 MTPA of LNG at its full capacity. By shutting down the nitrogen expander and adjusting the flowrates of the mixed refrigerant in the MCHE, the production capacity could be reduced, down to 5 MTPA. There are no technical limitations that restrict changing the production levels from 5 MTPA to 7.8 MTPA and vice versa [48]. However, operating at any production level between the two capacities is technically infeasible. Hence, an investor may either invest fully on a 7.8 MTPA AP-X train at the beginning of the project, or only on a 5 MTPA C3MR process, which could be expanded during the lifetime of the project by adding the nitrogen expander when the market develops.

In this step, the economic performance of the AP-X technology is evaluated deterministically under different demand scenarios and average selling prices, and stochastically under different demand scenarios considering the time-series behaviour of the HH natural gas spot prices using the MCS and mean-reverting GBM jump-diffusion process. Comparing the NPV with the ENPV enables the decision makers to investigate the impact of the uncertainties involved in the project when considering the volatility of markets and diversified selling strategies.

#### 5.3.1 Option a: taking the decision to not increase the capacity

Fig. 6(a)–(c) illustrates the NPV distribution for the three multi-demand scenarios in the high-income Asia Pacific markets. The scenarios comprise of the demand combinations of 5 MTPA, 6 MTPA, and 7.8 MTPA. However, due to the technical constraints, when there is a demand of 6 MTPA in the high-income Asia Pacific markets, the decision maker decides to produce only the contracted capaciy of 5 MTPA that it sold at a fixed price and to not cover the incremental increase in demand in the last 5 years of scenario 1, and the last 3 years of scenario 3. In scenario 1 the deterministic NPV was calculated as $2.68 billion, whilst the simulation of scenario 1 in Fig. 6(a) results in an NPV distribution with an ENPV of $2.88 billion. Moreover, the distribution has a median value of $2.83 billion and a standard deviation of $0.36 billion. In fact, the main attribute that impacts the NPV distribution in these scenarios is the volatility of the simulated HH natural gas prices in the first 10 years of production. A ENPV less than deterministic NPV indicates that HH prices decline below the initial price at year 2025, and, hence, reflects the volatile behavior of HH prices throughout the project's lifetime. Whilst in scenario 2 in Fig. 6(b), the NPV distribution is mainly impacted by the returns from selling LNG on the spot HH prices from the sixth year of production towards the end of the project's lifetime, resulting in an ENPV of $2.73 billion which is less than the deterministic NPV of $3.35 billion. Moreover, in this scenario, the median NPV is $2.63 billion with a standard deviation of $0.54 billion. A higher standard deviation in scenario 2 than scenario 1 implies a greater spread of values away from the median value. However, the decision maker in this case expands the project to its full capacity from the sixth year of production until the end of the project's lifetime to meet the increase in demand in Asia-Pacific markets. Lastly, the simulation outcomes of scenario 3 illustrated in Fig. 6(c) indicates an ENPV of $2.82 billion, 11% greater than the NPV calculated deterministically which is $2.54 billion, hence, satisfying Jensen's law and reflecting the opportunities that could be captured when considering investing in such a scenario which could not be identified when an average HH natural gas spot price was considered for NPV calculations. The economic performance of scenario 3 is mainly influenced by the simulated HH prices using MCS from year 4 of production until year 16.

#### 5.3.2 Option b: taking the decision to increase the capacity

In the second option, the decision maker decides to operate at fully capacity in the last 5 and 4 years of scenarios 1 and 3, respectively, to meet the slight increase in demand in Asia Pacific spot markets by selling at prices indexed to the HH spot pricing system whilst selling the remaining 1.8 MTPA produced LNG capacity to low-income markets at a fixed price of $3.00/MMTBU. Hence, compared to option 1, the NPV simulations in scenarios 1 and 3 are mainly influenced by the volatility of the simulated HH prices using the mean reverting GBM jump diffusion model and MCS in the last years of production. Moreover, the attractiveness of operating at full capacity and selling at a low fixed price to low-income markets was assessed.

**Fig. 7:**
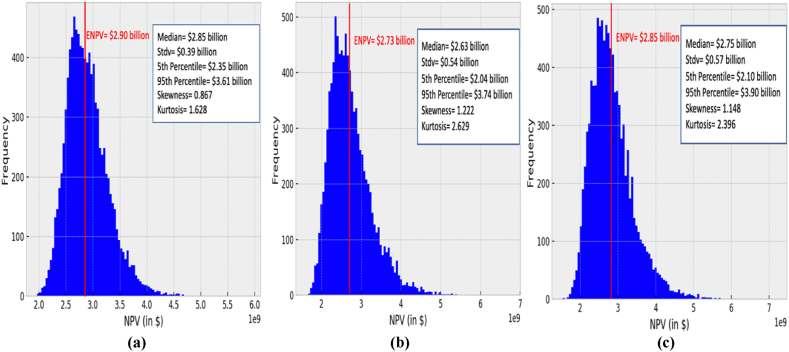
NPV distribution of an AP-X technology with initial forecasted Henry Hub natural gas spot price of $3.06/MMBTU under different demand scenarios and a diversified selling strategy (Option 2: deciding to increase the capacity and sell the remaining to low-income markets): (a) scenario 1, (b) scenario 2, and (c) scenario 3.

Fig. 7(a)-(c) illustrates the NPV distribution for the three demand scenarios in the high-income Asia Pacific markets and low-income markets as secondary markets. The simulation of scenario 1 in Fig. 7(a) results in an NPV distribution with an ENPV of $2.90 billion, which is approximately 2% less than the deterministic NPV calculated as $2.97 billion when considering the average HH natural gas spot price. Moreover, the distribution has a median value of $2.85 billion and a standard deviation of $0.39 billion. On the other hand, the simulation of scenario 3 illustrated in Fig. 7(c) results in an ENPV of $2.85 billion, which is 5.6% greater than the NPV calculated deterministically at $2.70 billion, hence, satisfying the Jensen's law. The difference between the calculated ENPVs of scenarios 1 and 2 in option 2 are quite greater than the ENPVs calculated when considering to not operate at full capacity in the last years of production in option 1. In fact, the impact of the stochastic HH prices of the last years of production is greater as the simulated series of prices become more divergent as moving towards the end of the project's lifetime, indicating greater uncertainties. On the other hand, an investor could prefer sticking to option 2 to increase the market share. However, different parameters such as the standard deviation, kurtosis values could be also considered when comparing options.

To assess the performance of a scenario, the standard deviation provides information about the spread of the simulated NPVs. A lower standard deviation value in scenario 1 than scenario 3 indicates a smaller spread of simulated NPVs - meaning that most of the values are concentrated near the median value of the distribution. Additionally, when analysing scenarios, one could also consider both tails of an NPV distribution, wherein a higher right tail indicates high market conditions that can be utilised, whilst a heavy left tail reflects the risks associated with a selling strategy. In this case, a positive skewness value indicates heavier right tails, whilst a negative skewness value indicates that the distribution has a heavier left tail. As noticed in Fig. 6, Fig. 7, the NPV distribution of all the three scenarios for both analysed options are skewed to the right with heavier right tails, indicating higher probability of profitable opportunities associated with the right tail.

Additionally, the parameter kurtosis measures the heaviness of both tails relative to the rest of the distribution. A kurtosis value greater than 3 implies heavier tails compared to a normal distribution and indicates the highly profitabile opportunities associated with the right tail, and the high risks associated with the left tail of the distribution. Consequently, an investor could further analyse both tails of a distribution using tail risk management by focusing on strategies to capture high market conditions within the right tail, and to minimise risks associated with the left tail. Although tail risks have a small probability, most investors are concerned with hedging against such rare events when the returns deviate from the mean, particularly by three standard deviations. Hence tail risk management strategies, such as put options are the most common for the tail-risk hedging. However, dealing with “real” options requires an in-depth analysis and was not considered as part of this study.

Overall step 3 indicates that scenarios 1 and 3 in both options demonstrate better economic performance due to having the ENPVs close to deterministic NPVs when considering a diversified selling strategy as an overall hedging strategy against the stochastic behaviour of HH natural gas spot prices. The differences in ENPVs between the two options are small. However, scenario 1 has the lowest positive skewness value, thus, implies lower probabilities for higher profitability in the right tail of the distribution compared to scenario 3 in option 1. Additionally, the skewness value further decreased by 4% in the second option influenced by the price simulation values in the last 5 years of production. Meanwhile, the kurtosis value increased slightly, by 1% only. This indicates that a higher ENPV can be achieved with a greater possibility for positive rare events rather than risks. Whilst in scenario 3, the skewness value has increased by only 0.3%, with a greater decrease in the kurtosis value by approximately 5%; indicating that the tails are getting flatter with lower probability of rare events at the tails.

### 5.4 Step 4: sensitivity analysis

**Fig. 8:**
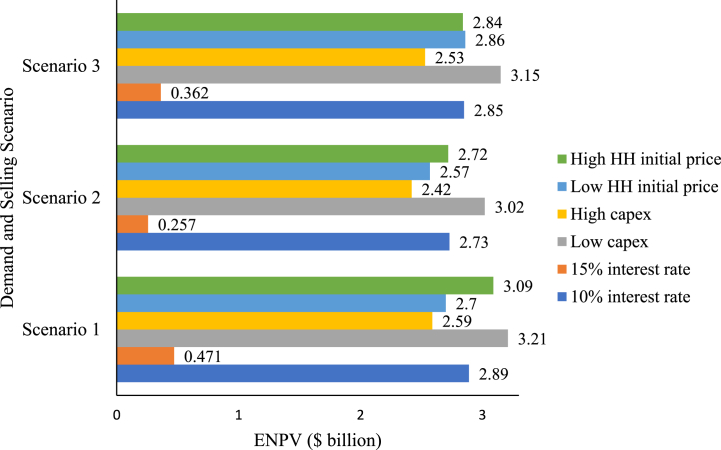
Evaluated ENPV of the three scenarios under varied capital costs, initial Henry Hub natural gas spot price, and interest rate.

The robustness of the proposed diversified selling strategy for hedging risks was tested under different conditions, such as different capex values of $660/tpa and $760/tpa; a constant interest rate of 15%, and an initial HH natural gas spot prices of $2.75/MMBTU and $3.06/MMBTU (with 10% of error). Fig. 8 illustrates the results of the ENPV sensitivity analyses for the three scenarios. Whilst Table 1 in the Appendix summarises the changes in standard deviation, skewness, and kurtosis under the same changing variables. As noticed in Fig. 8, the ENPV in all scenarios decreases when increasing the interest rate, increasing the capex value, or reducing the natural gas spot price. However, scenario 3 showed inconsistency in the trend when increasing the initial HH forecasted price by 10% relative to the baseline forecasted price of $3.06/MMBTU, wherein the ENPV decreased by 0.4% rather than exhibiting the same incremental trend examined in scenarios 1 and 2. Additionally, the fluctsuation in the ENPV subject to a ± 10% change in HH initial price in scenario 3 is minimal compared to other scenarios. Hence, indicating its robustness to changes in HH prices. Scenario 3 showed the lowest changes in ENPV at different input parameters compared to the other scenarios, with the greatest robustness examined when changing the HH initial price.

On the other hand, when focusing on the NPV distribution of the scenarios, the skewness and kurtosis changed differently with the changes in input conditions. In relative terms, when considering the shape of the NPV distribution, scenario 2 achieved the greatest skewness and kurtosis values at different input parameters. Incidentally, scenario 2 is the most skewed to the right with the heaviest right and left tails compared to other scenarios at varied input parameters. Consequently, an investor may consider strategies that optimise utilising high profitability opportunities associated with the right tail of the distribution, whilst considering strategies to manage the risks associated with the left tail of the distribution.

Overall, the evaluated approach provides a quantitative baseline assessment to support policymakers in deciding on embedding flexibility in the early design stages of LNG projects. The analysis indicates that by embedding flexibility in production, the decision makers would be more responsive to changes in the market through changing production capacities and targeting new markets during supply disruptions such as during wars. Hence, adding more liquidity to the global LNG trade.

## 6 Conclusion

In this study, a four-step methodological framework was proposed to assess the economic value of flexibility of an LNG production technology as an active mode of response to uncertainties in the high-income Asia Pacific markets. This analysis links flexible production system with market uncertainties by utilising the concepts from financial engineering in production to diversify sales and hedge against uncertainties. The outcomes of the analysis were as the following:

(i) The AP-X technology was selected and economically evaluated under deterministic and stochastic Henry Hub natural gas spot prices using Monte Carlo Simulation with the mean-reverting Geometric Brownian Motion Jump diffusion process.
(ii) The AP-X technology was further technically and economically evaluated under demand uncertainties by considering multi-demand scenarios in the high-income Asia Pacific markets throughout the lifetime of the project.
(iii) The demand scenarios assumed three-demand levels of 5,6, and 7.8 MTPA in the high-income Asia Pacific markets throughout the lifetime of the project. The decision-maker holds the option to increase the capacity or produce contracted capacities based on the market performance.
(iv) Diversified selling strategies comprised of selling under long-term contracts, short/mid-term contracts, and spot selling were assessed for the utilisation of market conditions and ability to hedge against the risks.
(v) The economic performance of the scenarios was mainly influenced by the simulated Henry Hub natural gas spot prices during the production years which impacted the results of the two studied options.
(vi) The sensitivity of the diversified selling strategy to changing input conditions was analysed. Increasing the interest rate to 15% had the greatest impact on the NPV distribution and the economic performance of the three scenarios.

The approach proposed in this study enables decision makers to understand how a flexible LNG production system can be employed to deal with uncertainties. Additionally, the strategy can be considered at different lifetimes of the project for backtesting when an abnormal event occurs to evaluate the influence of different decisions on the profitability of the project. The deterministic and stochastic analyses emphasises the influence of contractual structures on the economic performance of the project. Hence, contracting the full capacity of the project under LTCs is the most secure and probable profitable strategy for the producers. On the other hand, with the hesitance of importers to sign LTCs, the possible worst-case scenario would be to dedicate the full capacity to be sold based on HH natural gas spot prices. However, this scenario represents a great loss to the investors. A hybrid selling strategy is needed to hedge against the risks and diversify the selling strategy to be implemented by employing flexible production within a system, wherein the producer holds the option to increase the capacity or produce only contracted LNG based on the market conditions.

One major limitation of the current study is the assumption of fixed-level demand scenarios based on technological limits, instead of forecasting the possible future demand in the decision framework due to insufficient historical data. Future work could usilise time series models, such as Autoregressive integrated moving average (ARIMA) and Co-Integration for forecasting time-series prices and demand simultaneously. Additionally, only the time-series characteristics of the HH natural gas spot prices were considered for LNG spot pricing in the high-income markets. Future studies could also consider the stochastic behavior of utility prices and investigate a diversified selling strategy in multiple international markets considering the time-series behavior of diverse pricing systems, including Henry Hub, Title Facility Transfer, and National Balance Point. While the AP-X technology was assessed in this study, future studies could include assessing the value of flexibility for the POCP technology since it is the second most common technology worldwide. Moreover, for LNG projects in larger countries, various centralised and decentralised designs could be analysed to investigate the most optimal design configuration in terms of the project's profitability, considering the shipping costs and distances from the ports to different international markets.

## Author contribution statement

Rajesh Govindan, Luluwah Al-Fagih, Tareq Al-Ansari: conceived and designed the experiments; analysed and interpreted the data; contributed reagents, materials, analysis tools or data. Noor Yusuf: conceived and designed the experiments; performed the experiments; analysed and interpreted the data; contributed reagents, materials, analysis tools or data; wrote the paper.

## Data availability statement

Data included in article/supp. material/referenced in article.

## Additional information

No additional information is available for this paper.

## Declaration of competing interest

The authors declare that they have no known competing financial interests or personal relationships that could have appeared to influence the work reported in this paper.

## Figures and Tables

**Table 1:** Sensitivity analysis under variable input parameters for scenarios 1, 2, and 3

	Scenario 1		Scenario 2		Scenario 3	
	Increasing the interest rate					
	r = 10%	r = 15%	r = 10%	r = 15%	r = 10%	r = 15%
ENPV (x10^9^ $)	2.89	0.471	2.73	0.257	2.85	0.362
Stdev (x10^9^ $)	0.386	0.228	0.543	0.260	0.566	0.295
Skewness	0.867	0.883	1.222	1.318	1.148	1.073
Kurtosis	1.628	1.517	2.629	3.051	2.396	2.332
	**Increasing and decreasing capex by 7.5%**					
	**Low Capex**	**High Capex**	**Low Capex**	**High** **Capex**	**Low Capex**	**High Capex**
ENPV (x10^9^ $)	3.21	2.59	3.02	2.42	3.15	2.53
Stdev (x10^9^ $)	0.392	0.386	0.535	0.542	0.567	0.564
Skewness	0.966	0.932	1.379	1.347	11.067	1.124
Kurtosis	1.867	1.498	4.442	3.179	2.003	2.424
	**Increasing and decreasing the initial HH forecasted price by 10%**					
	**Low HH price**	**High HH price**	**Low HH price**	**High HH price**	**Low HH price**	**High HH price**
ENPV (x10^9^ $)	2.70	3.09	2.57	2.72	2.86	2.84
Stdev (x10^9^ $)	0.351	0.428	0.483	0.584	0.505	0.613
Skewness	0.927	0.882	1.381	1.245	1.072	1.125
Kurtosis	1.906	1.364	3.946	2.682	2.000	2.272
